# Multidisciplinary Management of Bilateral Ureteric Obstruction and Forniceal Rupture in Twin Pregnancy: A Case Report

**DOI:** 10.1002/ccr3.71163

**Published:** 2025-10-04

**Authors:** Ernest Kwame Adjepong‐Tandoh, Yaw Boateng Mensah, Michael Yao Ntumy, James Edward Mensah

**Affiliations:** ^1^ Department of Surgery Korle Bu Teaching Hospital Accra Ghana; ^2^ Department of Radiology University of Ghana Medical School Accra Ghana; ^3^ Department of Obstetrics and Gynaecology University of Ghana Medical School Accra Ghana; ^4^ Department of Surgery University of Ghana Medical School Accra Ghana

**Keywords:** acute abdominal pain in pregnancy, bilateral ureteric obstruction, forniceal rupture, percutaneous nephrostomy, resource‐limited setting, twin pregnancy

## Abstract

Forniceal rupture during twin pregnancy is rare and mimics other abdominal emergencies such as ureteric colic, appendicitis, and ruptured ectopic; therefore, diagnosis requires a high index of suspicion. A multidisciplinary approach involving obstetricians, urologists, radiologists, general surgeons, and individualized intervention is key to achieving good maternal and fetal outcomes.

## 1 Introduction

Renal forniceal rupture, characterized by the leakage of urine from the renal calyces due to increased pressure in the renal collecting system, often results from urolithiasis [1] but can also arise from malignancy, infection, iatrogenic injury, or, less commonly, pregnancy [2]. Patients typically present with vague flank pain, which can resemble an acute surgical abdomen, leading to potential misdiagnosis and unnecessary surgery [3, 4, 5]. In pregnancy, untreated forniceal rupture is associated with serious complications such as preterm birth and acute kidney injury [4, 6]. Accurate diagnosis is challenging, requiring a high index of suspicion, especially when immediate radiologic or surgical resources are unavailable. This report details the management of a 31‐year‐old primigravida with a 27‐week twin gestation who developed bilateral ureteric obstruction and right forniceal rupture, necessitating bilateral percutaneous nephrostomies and resulting in preterm labor. The case underscores the importance of multidisciplinary management and contributes to the limited literature on this rare condition during pregnancy.

## 2 Case History and Examination

We report the case of a 31‐year‐old primigravida, a Ghanaian (African) and a regular antenatal clinic attendee with no significant medical history, who presented at the obstetric emergency room at 27 weeks and 2 days of twin gestation with a 2‐h episode of acute right flank pain. The pain was non‐positional, rated 8 out of 10 in severity, and radiated to the right iliac fossa. She had vomited twice and denied any history of trauma, fever, chills, or previous urolithiasis. Physical examination was unremarkable except for mild tenderness over the right renal angle and right iliac fossa.

## 3 Differential Diagnosis

Ureteric colic, acute appendicitis, acute cholecystitis.

## 4 Investigations and Treatment

**FIGURE 1:**
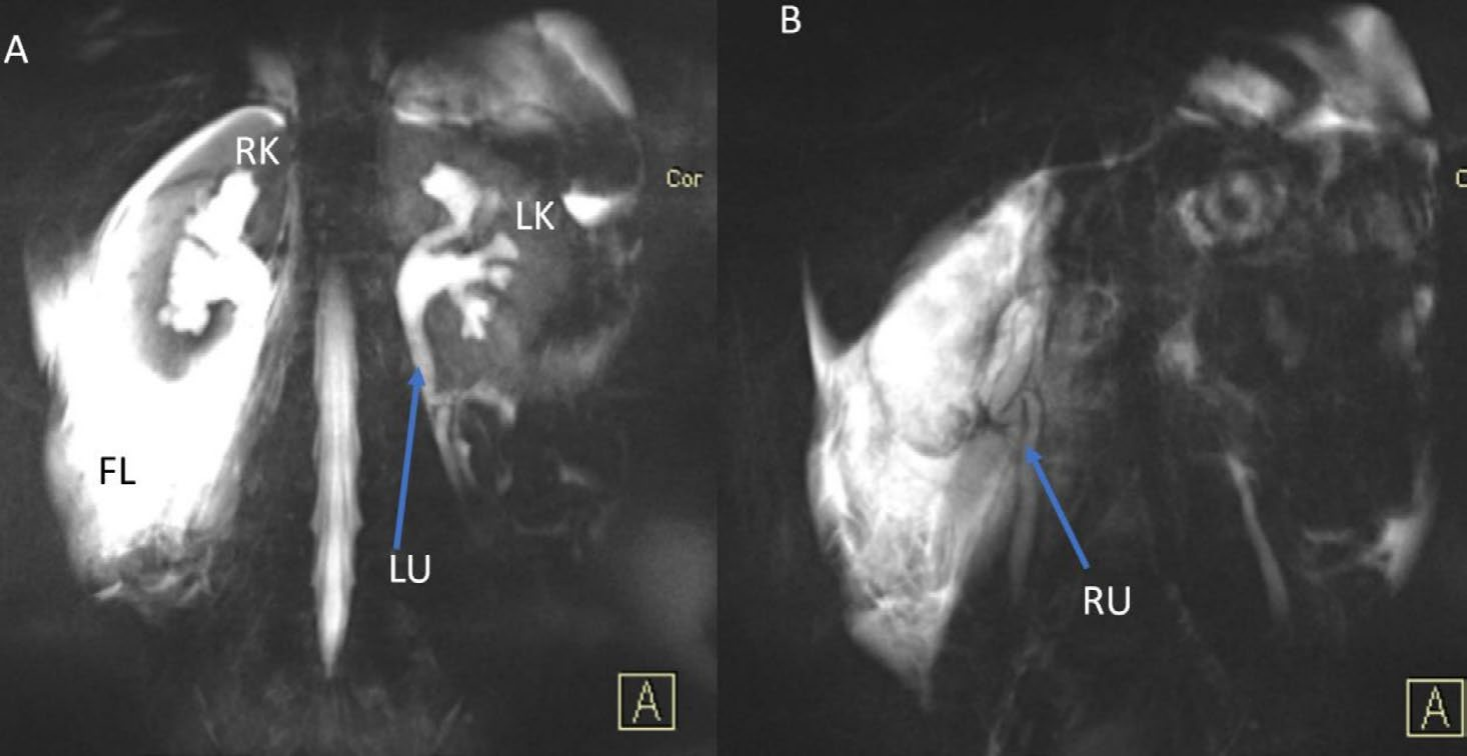
(A) Coronal fat suppressed T2W image showing moderate calyceal dilatation in the right kidney (RK) and mild calyceal dilatation in the left kidney (LK). There is significant fluid (FL) around RK. The left ureter (LU) shows a kink at the junction between the proximal and distal third of the ureter. (B) 3D image shows severely kinked right ureter (RU).

**FIGURE 2:**
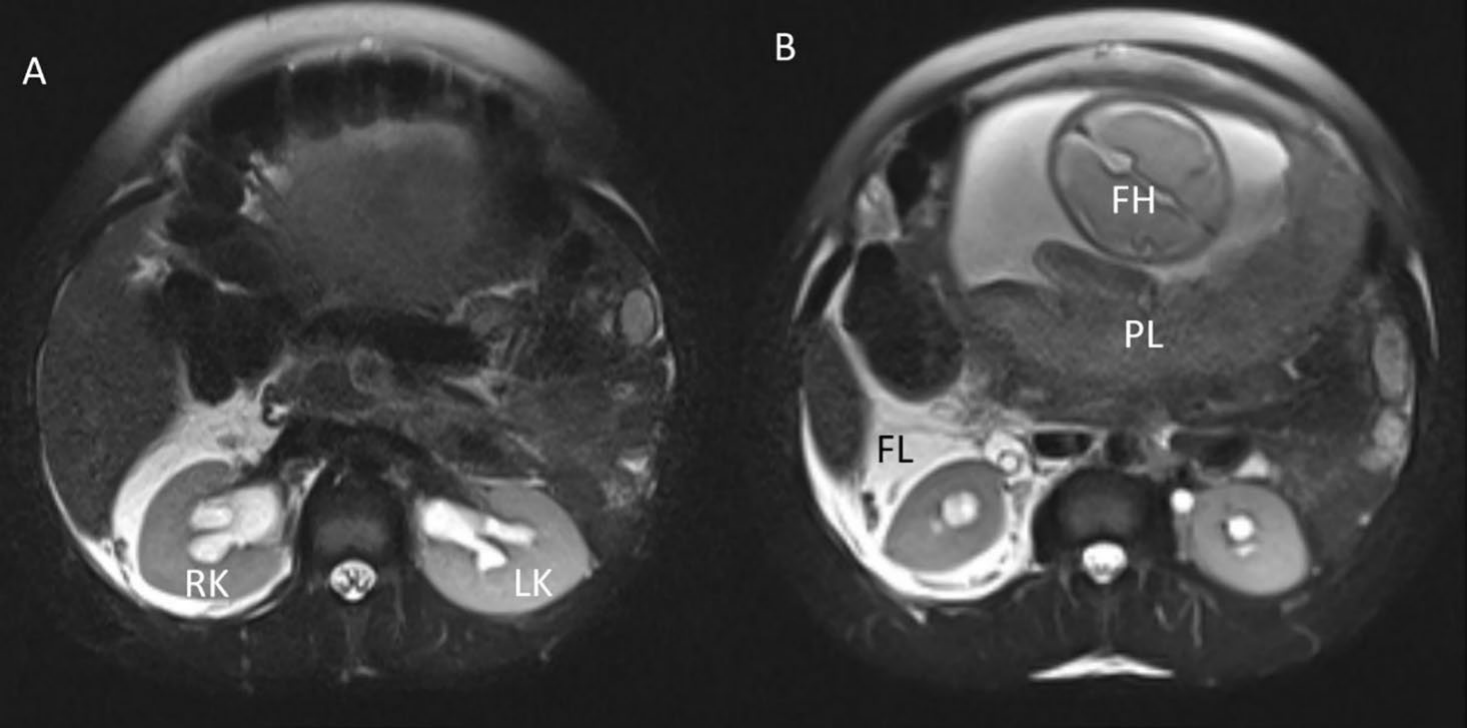
Axial T2W MRI images of the abdomen at the level of the kidneys. (A, B) show bilateral calyceal dilatation, with fluid (FL) around the right kidney (RK). (B) shows part of the gravid uterus with a fetal head (FH).

**FIGURE 3:**
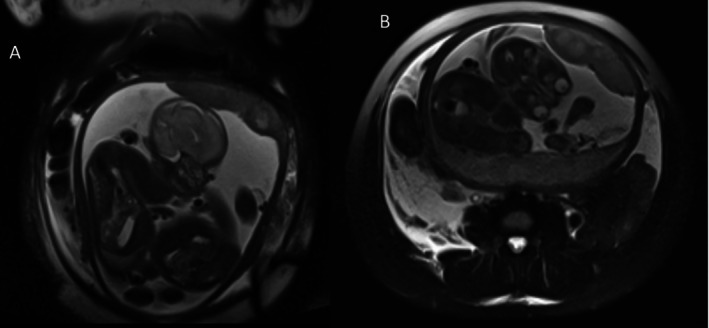
Coronal (A) and Axial (B) T2W MRI images showing a gravid uterus with twins and moderate free peritoneal collection.

An abdominal‐pelvic ultrasound (USG) showed mild‐to‐moderate right hydroureteronephrosis with a right perirenal collection and mild left hydronephrosis, with no signs of an inflamed appendix. An MRI scan of the abdomen corroborated these findings (Figures 1, 2, 3). No evidence of calculi was noted bilaterally in the upper and mid‐ureters; the distal ureters were not visualized on the MRI.

On the third day post admission, an ultrasound‐guided aspiration of the perirenal collection yielded amber‐colored fluid with an aspirate‐to‐serum creatinine ratio of 16.8, confirming extravasation of urine into the retroperitoneum from a right forniceal rupture secondary to distal ureteric obstruction.

The patient continued to experience persistent right flank pain, which was not responsive to IV paracetamol, and a deterioration in glomerular filtration rate (GFR) was noticed. A percutaneous right perirenal drainage tube was inserted on Day 4. The pain subsided considerably, but the tube continued to drain about 1800 mL of urine daily, which we thought could be due to distal ureteric obstruction since the distal third of the ureter was not visualized on the MRI. Therefore, an obstructing distal ureteric calculus could not be excluded. We were concerned that if a distal obstruction was present, it had to be relieved to stop the urine extravasation from the forniceal rupture. We thought that the gravid uterus could not be responsible for such a high‐grade obstruction.

The importance of conclusively excluding a stone as the primary cause of the ureteric obstruction with a low‐dose pelvic CT scan was discussed with the patient, but she declined due to her fear of exposing her baby to any form of radiation. Due to persistent perirenal drainage, a rise in white blood cell count, and low‐grade fever, we suspected a possible infection of the perirenal collection. We, therefore, placed a right percutaneous nephrostomy tube (PCN) and passed a urethral catheter for continuous bladder drainage.

The perirenal drain output ceased 3 days after the PCN was inserted, and renal function and WBC count normalized with intravenous antibiotics (Meropenem, then Ceftriaxone). She was discharged after 17 days of admission, with explicit instructions for nephrostomy care and a chart to record urine output from both the PCN and per urethra. Fetal lung maturation was induced with IM dexamethasone prior to discharge. The obstetric team planned a cesarean section at 37 completed weeks.

Ten days after discharge, she was readmitted, presenting with no urine output from the urethral catheter for 24 h, but with normal drainage from the right nephrostomy tube. An abdominal and pelvic ultrasound revealed severe left hydronephrosis, warranting the immediate placement of a left percutaneous nephrostomy (PCN). We, therefore, concluded that the twin gestation was compressing the pelvic ureters, which had caused complete bilateral ureteric obstruction and right forniceal rupture. The plan was to maintain both nephrostomy tubes until the neonatologist deemed the fetal maturity adequate. However, she went into labor only 3 h after insertion of the second nephrostomy. She gave birth to two live preterm male infants at 32 weeks and 2 days of gestation via spontaneous vaginal delivery. Both infants had a cephalic presentation and weighed 1.9 and 1.7 kg at birth. Both had Apgar scores of 7 and 8 at 1 and 5 min, respectively.

## 5 Outcome and Follow‐Up

She started passing urine per urethra immediately after delivery. The PCN tubes were clamped and subsequently removed after no flank pain was noted, and renal ultrasound showed markedly diminished hydronephrosis bilaterally. Both neonates were admitted to the Neonatal Intensive Care Unit due to their prematurity, with good clinical outcomes.

## 6 Discussion

This case report of a twin pregnancy leading to bilateral ureteric obstruction and forniceal rupture underscores the diagnostic and therapeutic complexities associated with this rare condition. Diagnosing renal forniceal rupture in pregnancy is particularly challenging. While flank pain or costovertebral angle tenderness may suggest this diagnosis in non‐pregnant patients, the physiological changes of pregnancy can obscure these symptoms [4]. For instance, the displacement of the appendix by a gravid uterus can lead to atypical presentations of appendicitis, and similarly, the physiologic dilation of the ureters during pregnancy complicates the identification of ureteric obstructions [7]. To avoid unnecessary interventions and adverse outcomes, clinicians must be more aware and carefully consider a broad differential, along with appropriate imaging and multidisciplinary management.

During pregnancy, physiological and hormonal changes, such as the enlargement of the kidneys and ureteral compression by the expanding uterus, increase the risk of forniceal rupture [2]. These changes, along with the effects of hormones such as progesterone and prostaglandins, cause ureteropelvic distension, heightening the likelihood of rupture. Compression of the ureters by the gravid uterus is a key factor, particularly on the right side, where 88% of ruptures occur in pregnant patients compared to 49% in non‐pregnant patients [8, 9]. This anatomical predisposition is likely due to the course of the ovarian veins, with the right‐sided vein crossing the ureter to enter the vena cava, increasing the risk of obstruction. In contrast, the left‐sided vessel runs in parallel with the ureter and enters the renal vein [8].

The clinical presentation of forniceal rupture during pregnancy can closely resemble various acute abdominal conditions; it is crucial to conduct a prompt and thorough evaluation, focusing on both maternal stability and fetal well‐being. Differential diagnoses should consider obstetric and gynecologic conditions such as placental abruption, preterm labor, ectopic or heterotopic pregnancy, ovarian cysts or tumors, pelvic inflammatory disease, and fibroid degeneration [5]. Renal and urologic conditions, including nephrolithiasis, pyelonephritis, spontaneous forniceal rupture, and renal malignancy, should also be considered, alongside gastrointestinal and hepatobiliary conditions such as appendicitis, pancreatitis, gallbladder disease, inflammatory bowel disease, and bowel obstruction [2]; therefore, not surprisingly, a common cause of harm in forniceal rupture cases stems from incorrect initial diagnoses leading to unnecessary surgeries. For instance, in a report in 2016, appendectomies were mistakenly performed in 17% of cases [10]. Laboratory tests often fail to provide diagnostic information, making imaging a critical component of the diagnostic process.

Ultrasound and MRI are typically employed in the diagnostic workup of pregnant patients, with ultrasound often being the first‐line investigation. Ultrasound has proven effective in diagnosing urinary extravasation from the renal pelvis in more than half of the reported cases [4]. It also offers the additional benefits of identifying hydronephrosis, detecting calculi, and assessing fetal well‐being, making it a valuable tool for managing pregnant patients presenting with flank pain. In cases where ultrasound does not immediately confirm the diagnosis, a repeat scan may be warranted. MRI is the next recommended imaging modality when ultrasound fails to diagnose forniceal rupture, and it has been successful in diagnosing spontaneous renal forniceal rupture in approximately 75% of cases [8]. In low‐resource settings where MRI may not be available, other imaging modalities, such as low‐dose retrograde pyelograms, could be considered. Although retrograde pyelography has become less common due to advancements in imaging technology, it may still be useful if MRI is not available or affordable. Computed tomography (CT) scans, even with low‐dose protocols, should generally be avoided during pregnancy due to the potential risks of ionizing radiation to the fetus [11]. This highlights the importance of individualized decision‐making and the need for imaging strategies that balance diagnostic accuracy with safety considerations for both the mother and fetus.

Managing forniceal rupture during pregnancy is complex and requires a tailored approach based on the severity and location of urine leakage. Treatment options include ureteric stents, percutaneous drains, conservative management, and nephrostomy tube placement. In this case, a perirenal drain was initially used to manage the urinoma, but persistent high drainage and the risk of infection necessitated the placement of percutaneous nephrostomy tubes. Conservative management can be an option as pregnancy nears full term, particularly in uncomplicated cases with minimal pain, but it may not always succeed. For example, Boekhorst reported a case where conservative management failed due to ongoing pain, leading to the placement of ureteral stents, which promptly resolved symptoms [3]. Bilateral ureteric stenting without fluoroscopic guidance can be risky due to the potential for misplacement in the perirenal space. Additionally, stents in pregnant patients require frequent changes due to a heightened risk of encrustation, likely related to pregnancy‐induced hypercalciuria and hyperuricosuria [12]. When available, ultrasound‐guided stent placement can reduce radiation exposure, though pulsed fluoroscopy is an alternative if necessary [13]. Nephrostomies, while effective, increase infection risk and require periodic changes to minimize complications. The physical and emotional discomfort associated with bilateral nephrostomy tubes in a pregnant patient is significant, affecting sleep, daily activities, and overall quality of life [12]. The risk of infection or accidental dislodgement adds to these challenges, underscoring the importance of patient education and support.

Monitoring for obstruction in the contralateral kidney and intervening promptly is critical. The decision to maintain nephrostomy tubes until fetal maturity must carefully weigh maternal comfort against the risk of preterm delivery [8]. In some cases, early delivery can alleviate the obstruction caused by the gravid uterus, potentially eliminating the need for further urological interventions [4]. A key consideration in such cases is balancing fetal maturity and survival against the risks to the mother. In well‐equipped medical settings, neonatologists might recommend early delivery if maternal complications are severe, as neonatal intensive care units (NICUs) can provide the necessary support for premature infants. However, in resource‐limited environments, the ability to care for premature babies may be restricted. This makes it crucial to delay delivery as long as possible to improve the baby's chances of survival. Administering medications such as intramuscular dexamethasone to help the fetus's lungs mature can also be beneficial if early delivery becomes necessary [4].

The patient went into premature labor, delivering 3 h after the insertion of the second nephrostomy tube. While there are not enough documented cases of forniceal rupture to definitively establish a link with preterm birth [14], the connection is plausible. Inflammation, tissue injury, and irritation of the peritoneum caused by urine leakage could potentially trigger preterm labor. More research is needed to explore whether there is a direct correlation between forniceal rupture and preterm birth and to identify any underlying physiological mechanisms.

## 7 Conclusion

This case highlights a rare occurrence of spontaneous forniceal rupture in a twin pregnancy due to bilateral ureteric obstruction. It demonstrates how the condition can mimic other causes of acute abdomen in pregnancy, leading to diagnostic uncertainty. Successful management relied on high clinical suspicion, appropriate imaging with MRI and ultrasound, and timely intervention using bilateral percutaneous nephrostomies. The case underscores the value of multidisciplinary collaboration in achieving favorable maternal and neonatal outcomes, especially in resource‐limited settings.

## Author Contributions

**Ernest Kwame Adjepong‐Tandoh:** conceptualization, data curation, formal analysis, investigation, writing – original draft, writing – review and editing. **Yaw Boateng Mensah:** conceptualization, investigation, writing – original draft, writing – review and editing. **Michael Yao Ntumy:** conceptualization, investigation, writing – original draft, writing – review and editing. **James Edward Mensah:** conceptualization, data curation, formal analysis, investigation, supervision, writing – original draft, writing – review and editing.

## Ethics Statement

The authors have nothing to report.

## Consent

Written informed consent for the publication of this anonymised case report and any accompanying images was obtained from the patient. All data has been de‐identified to protect the privacy of the patient. A copy of the written consent is available for review by the editor‐in‐chief of this journal upon request.

## Conflicts of Interest

The authors declare no conflicts of interest.

## Data Availability

The authors have nothing to report.
